# Transplant Trial Watch

**DOI:** 10.3389/ti.2024.14062

**Published:** 2024-11-28

**Authors:** John M. O’Callaghan, Simon R. Knight, Reshma Rana Magar

**Affiliations:** ^1^ Centre for Evidence in Transplantation, Nuffield Department of Surgical Sciences, University of Oxford, Oxford, United Kingdom; ^2^ University Hospitals Coventry and Warwickshire, Coventry, United Kingdom; ^3^ Oxford Transplant Centre, Churchill Hospital, Oxford, United Kingdom

**Keywords:** randomised controlled trial, systematic review/meta-analysis, kidney transplantation (KT), heart transplantation, hypothermic oxygenated machine perfusion

## Aims

This study aimed to compare the outcomes associated with different classes and combinations of antihypertensive drugs in renal transplant patients.

## Interventions

A literature search was conducted using the Cochrane Kidney and Transplant Register of Studies. Study selection and data extraction were performed by two independent reviewers. The risk of bias was assessed using the Cochrane risk of bias tool.

## Participants

97 studies were included in the review.

## Outcomes

The primary outcomes were death (all-causes), death-censored graft loss and kidney function. The secondary outcomes were cardiovascular death and other cardiovascular events, blood pressure, acute rejection, proteinuria, haemoglobin (Hb) and/or hematocrit (HCT), serum potassium and/or hyperkalaemia, infection, cancer, life participation, dementia, falls, fatigue, hypoglycemia and other adverse effects.

## Follow-Up

N/A.

## CET Conclusion

*by Reshma Rana Magar*

This systematic review looks at the benefits and harms associated with antihypertensive drugs in kidney transplant recipients. This is an updated version of the 2009 Cochrane review. A total of 97 studies were included, all of which were randomised controlled trials, apart from one which had a quasi-randomised design. Study selection, data extraction and quality assessment were performed independently by two reviewers. The GRADE approach was used to rate the certainty of evidence. The study found that, compared to standard care alone or placebo, calcium channel blockers (CCB) treatment significantly reduced all-cause death and graft loss, while angiotensin receptor blockers (ARB) was found to reduce graft loss. However, the certainty of evidence was moderate for CCB and low for ARB. Overall, the methodological quality of this paper is good and provides a granular analysis of the available data. Where heterogeneity was observed, attempts were made to explore it using subgroup analysis and meta-regression. However, for some of the outcomes, the number of studies included in the analyses were to low (1 or 2). Data were not analysed separately for living versus deceased donor transplants.

## Trial Registration

N/A.

## Funding Source

No funding received.

## Aims

This study aimed to compare short-term outcomes of continuous, hypothermic oxygenated machine perfusion (HOPE) versus static cold storage (SCS).

## Interventions

Donor hearts were randomised to either preservation with HOPE or SCS.

## Participants

229 adults (aged ≥18 years) in the waitlist for heart transplantation. Donors criteria were adults (age ≥18 to ≤70 years) accepted as a heart donor by the transplantation team.

## Outcomes

The primary endpoint was the time to first event of a composite measure (including graft failure, cardiac-related death, cellular rejection of at least grade 2R, moderate or severe primary graft dysfunction (PGD) of the left ventricle or PGD of the right ventricle). Secondary endpoints were the composite primary endpoint, duration of stay at the intensive care unit, cardiac injury markers, echocardiography data, incidence and duration of any postoperative mechanical circulatory support, incidence of major adverse cardiac transplant events (MACTE), and overall success or failure.

## Follow-Up

30 days post-transplantation.

## CET Conclusion

*by Simon Knight*

This multicentre RCT investigates the role of hypothermic oxygenated machine preservation (HOPE) of the DBD heart prior to transplantation. 229 patients were randomised to HOPE or static cold storage. 100 donor hearts underwent HOPE, and all were transplanted. The primary endpoint of cardiac-related death, graft dysfunction, rejection or graft failure was numerically lower in the study group (HR 0.56), but did not quite reach statistical significance (*p* = 0.059). Most of the difference in the primary endpoint appears to be driven by a significant reduction in risk of primary graft dysfunction with use of HOPE. The study is well-designed and well conducted, with an inclusive donor and recipient population reflective of clinical practice. Allocation concealment is good with centralised randomisation and stratification, and ITT analysis is used. Use of a complex primary endpoint with components of different severity is questionable, and outcomes were only measured for 30-day post-transplant. Whilst the primary endpoint is not quite met, the study provides compelling evidence that use of HOPE is safe in the short-term and can reduce the risk of primary graft dysfunction following DBD cardiac transplantation.

## Jadad Score

3.

## Data Analysis

Strict intention-to-treat analysis.

## Allocation Concealment

Yes.

## Trial Registration

ClinicalTrials.gov - NCT03991923.

## Funding Source

Industry funded.

## Clinical Impact Summary

*by John O’Callaghan*

This is a large and well-conducted RCT in heart transplantation comparing Hypothermic Oxygenated machine Perfusion (HOPE) to static cold storage. It was conducted across multiple transplant centres in 8 European countries. The methods of randomisation, data analysis and full follow up make the results reliable. The study builds on work in pre-clinical and clinical feasibility studies in heart transplantation using HOPE, as well as a now-considerable evidence base in the preservation of other organs.

The device used to deliver HOPE in this study was the XVIVO Heart Assist Transport (XVIVO Group, Gothenburg, Sweden) and primed with the cardioplegic solution from the same company, XVIVO Heart Solution, with additional recipient matched blood or erythrocytes, antibiotics, and insulin. This is a portable and automated device, taken to the donor centre so that the heart could be placed inside as soon as possible after retrieval. Of the 100 donor hearts preserved using the HOPE device, 3 were not transplanted, and this was for reasons unrelated to the device or preservation.

The primary outcome was a composite of cardiac-related death, specific grades of Primary Graft Dysfunction (PGD) or cellular rejection and early graft failure. There was a substantial reduction in this primary outcome associated with HOPE preservation (19% versus 30%, HR = 0.56) but with the statistical analysis plan this did not reach statistical significance in this study size (*p* = 0.59). The sample size was predicated on a 60% reduction in the primary endpoint, which may have been selected to achieve a reasonable study size and considering prior work. However, a reduction of 44% in the primary outcome, as seen here, would certainly be clinically significant. Also, there was a significant reduction in PGD (11% versus 28%) and severe PGD (5% versus 20%) when looked at alone. This is despite an overall longer median preservation time of hearts in the HOPE group (240 min versus 215 min).

This study clearly supports the use of HOPE for DBD cardiac allograft preservation compared to static cold storage. Further work should now be done to see if there is benefit in using HOPE to expand the potential donor pool, and if there is a role in DCD heart preservation.

### Clinical Impact Rating

5/5.

